# Grey and White Matter Magnetisation Transfer Ratio Measurements in the Lumbosacral Enlargement: A Pilot *In Vivo* Study at 3T

**DOI:** 10.1371/journal.pone.0134495

**Published:** 2015-07-31

**Authors:** Chinyere O. Ugorji, Rebecca S. Samson, Martina D. Liechti, Jalesh N. Panicker, David H. Miller, Claudia A. M. Wheeler-Kingshott, Marios C. Yiannakas

**Affiliations:** 1 NMR Research Unit, Queen Square MS Centre, Department of Neuroinflammation, UCL Institute of Neurology, London, United Kingdom; 2 Department of Uro-Neurology, The National Hospital for Neurology and Neurosurgery and UCL Institute of Neurology, London, United Kingdom; Heidelberg University Hospital, GERMANY

## Abstract

Magnetisation transfer (MT) imaging of the central nervous system has provided further insight into the pathophysiology of neurological disease. However, the use of this method to study the lower spinal cord has been technically challenging, despite the important role of this region, not only for motor control of the lower limbs, but also for the neural control of lower urinary tract, sexual and bowel functions. In this study, the feasibility of obtaining reliable grey matter (GM) and white matter (WM) magnetisation transfer ratio (MTR) measurements within the lumbosacral enlargement (LSE) was investigated in ten healthy volunteers using a clinical 3T MRI system. The mean cross-sectional area of the LSE (LSE-CSA) and the mean GM area (LSE-GM-CSA) were first obtained by means of image segmentation and tissue-specific (i.e. WM and GM) MTR measurements within the LSE were subsequently obtained. The reproducibility of the segmentation method and MTR measurements was assessed from repeated measurements and their % coefficient of variation (%COV). Mean (± SD) LSE-CSA across 10 healthy subjects was 59.3 (± 8.4) mm^2^ and LSE-GM-CSA was 17.0 (± 3.1) mm^2^. The mean intra- and inter-rater % COV for measuring the LSE-CSA were 0.8% and 2.3%, respectively and for the LSE-GM-CSA were 3.8% and 5.4%, respectively. Mean (± SD) WM-MTR was 43.2 (± 4.4) and GM-MTR was 40.9 (± 4.3). The mean scan-rescan % COV for measuring WM-MTR was 4.6% and for GM-MTR was 3.8%. Using a paired t-test, a statistically significant difference was identified between WM-MTR and GM-MTR in the LSE (*p*<0.0001). This pilot study has shown that it is possible to obtain reliable tissue-specific MTR measurements within the LSE using a clinical MR system at 3T. The MTR acquisition and analysis protocol presented in this study can be used in future investigations of intrinsic spinal cord diseases that affect the LSE.

## Introduction

Neurological disorders affecting the spinal cord (SC) can involve either the grey matter (GM), white matter (WM) or both. Conditions such as multiple sclerosis (MS), amyotrophic lateral sclerosis (ALS) and neuromyelitis optica affect both tissue types [1–3], whereas certain leukodystrophies affect the WM tracts only [4, 5]. Imaging tools that offer reliable assessment of tissue-specific (i.e. GM and WM) pathological involvement in the SC have potential to be helpful in the differential diagnosis, and also in monitoring the course and guiding the treatment, of such neurological conditions.

Magnetisation transfer (MT) imaging is a quantitative magnetic resonance imaging method (qMRI), which can be used in the central nervous system to study the interaction between restricted protons (i.e. protons bound to macromolecules) and free protons [6–8]. *Ex vivo* investigations have previously shown that the magnetisation transfer ratio (MTR) correlates with tissue myelin content and, less strongly, with axonal density in the brain [9] and spinal cord [10]. Normal regional variations in MTR have been reported in the adult brain [11–13], in normal aging [14] and the upper spinal cord [15, 16] *in vivo*. Significant reductions in MTR values, as compared to healthy controls, have been reported in the upper SC in cases of ALS [17], MS [18–20] and spinal cord injury (SCI) [21, 22], with significant correlations identified between the MTR measures and common measures of sensory and motor dysfunction.

Whilst MTR measurements in the upper SC have already provided insight into the pathophysiology of certain neurological conditions [17–22], to date there are no clinical studies reporting such changes in the lower SC. This region plays quite an important role not only for motor control of the lower limbs, but also for the neural control of lower urinary tract (LUT), sexual and bowel functions [23]. Recently, a new MRI method has been presented, which reliably identifies the widest cross-section of the lower SC (i.e. the lumbosacral enlargement; LSE) and proposes this as an intrinsic imaging biomarker in the study of neurological disease involving the SC [24]. Whilst the method has been shown to offer reliable GM and WM cross-sectional area (CSA) measurements within the LSE, its utility in terms of facilitating tissue-specific qMRI investigations such as MTR is yet to be explored.

In this pilot study, the feasibility of obtaining tissue-specific MTR measurements within the LSE was investigated for the first time by utilising the detailed structural information obtainable with the previously reported method [24], while at the same time addressing the technical challenges associated with MTR measurements at that level to provide a reliable and clinically viable index sensitive to myelin content in the lower SC.

## Materials and Methods

### Study Participants

Ten healthy volunteers (4 male and 6 female, mean age 28 years; range 26–35 years) were recruited for this study, with five of these returning for a repeat scan. Written informed consent was obtained from all study participants and the work was approved by the National Hospital for Neurology and Neurosurgery and the Institute of Neurology Joint Research and NRES committee London Bloomsbury (Formally, London REC2 Ethics Committee).

### MR Imaging

A 3T Philips Achieva MRI system with radiofrequency (RF) multi-transmit technology (Philips Healthcare, Best, Netherlands) and the manufacturer’s product 16-channel neurovascular coil and 15-channel SENSE spine coil were used. A 2D fast-spin echo (FSE) T2-weighted reference scan of the thoraco-lumbar spine was first obtained in the sagittal plane and used to facilitate planning of subsequent high-resolution structural and MTR acquisitions perpendicular to the longitudinal axis of the SC.

**Fig 1:**
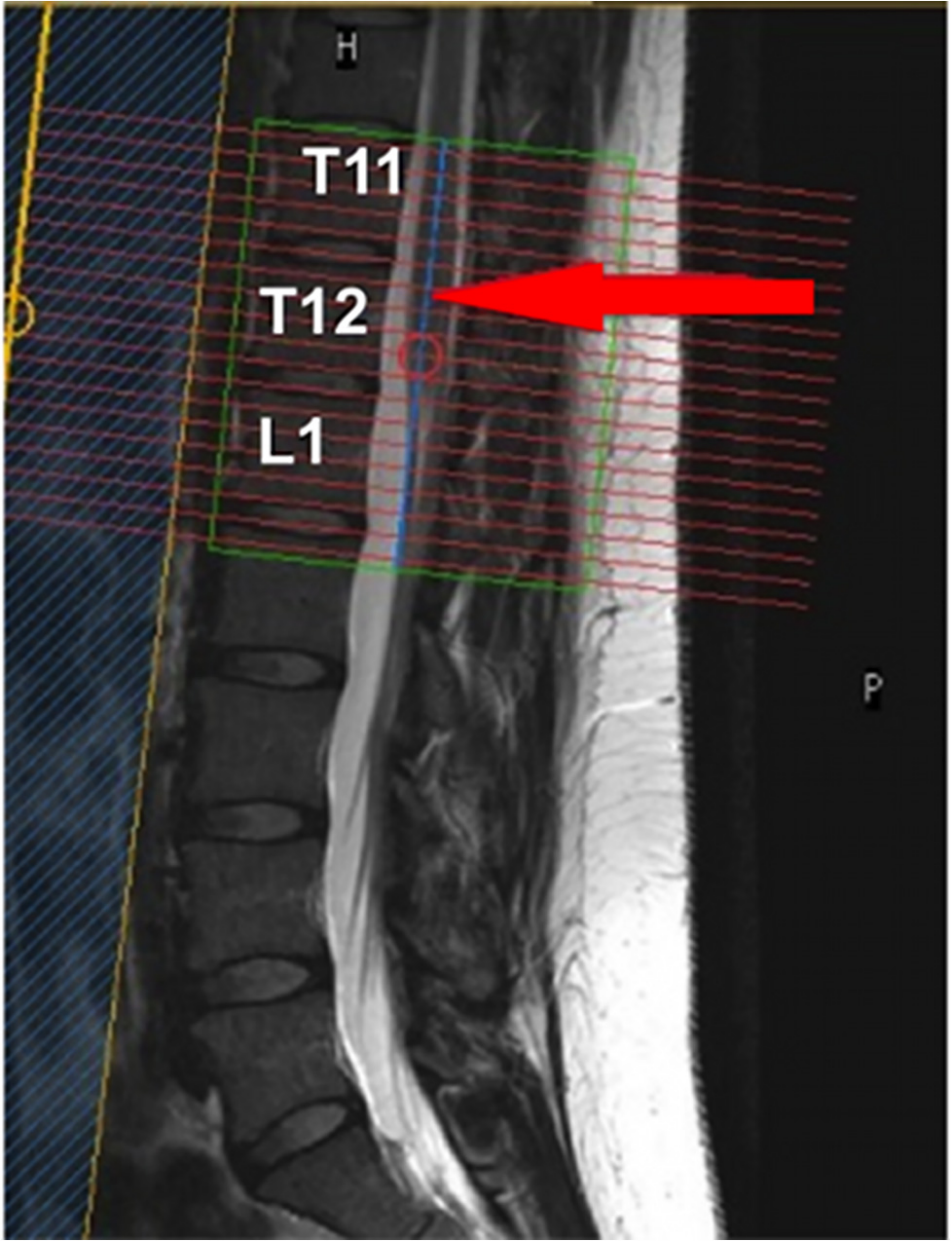
Prescription of the imaging volume between T11-L1 vertebral level to ensure coverage of the lumbosacral enlargement (LSE). Red arrow shows the position of the widest section of the LSE within the volume in this particular case.

For GM and WM segmentation within the LSE, a high-resolution acquisition was prescribed in the axial-oblique plane (i.e. perpendicular to the cord) through the T11—L1 vertebral level, to ensure coverage of the LSE in all cases as previously demonstrated [24, 25] (Fig 1). For this, a 3D slab-selective fast field echo (3D-FFE) sequence was used with fat suppression and the following imaging parameters: TR = 22 ms, TE = 4.4 ms, flip angle α = 10°, FOV = 180 x 180 mm; voxel size = 0.5 x 0.5 x 5 mm^3^; NSA = 8, slices = 19; slice gap = 0. The scan time for this acquisition was approximately 19 minutes.

MTR imaging was performed using identical scan geometry to the structural acquisition with the following parameters: 3D slab-selective FFE with two echoes (TR / TE1 / TE2 = 36 / 1.69 / 3.1 ms; flip angle α = 10°), with and without Sinc—Gaussian shaped MT saturation pulses with nominal α = 360°; offset frequency = 1 KHz, and duration 16 ms; number of slices = 45; FOV = 180 x 180 mm; acquisition matrix = 240 x 240 mm; voxel size = 0.75 x 0.75 x 5 mm^3^ (reconstructed voxel size in-plane = 0.5 x 0.5 mm). MTR data were acquired using a radial acquisition profile with the fold-over direction set in the foot/head (FH) direction [26]. The acquisition time for the MTR sequence was approximately 20 mins and the total scan time for the entire imaging protocol was approximately 45 mins.

Participants were positioned in the MRI scanner in such way to ensure close contact of the lower back with the flat surface of the spine coil to maximise the signal-to-noise ratio (SNR). This was achieved by elevating the legs using a triangular foam pad providing support under the knees; this ensured that the curvature of the lumbar spine was reduced thereby minimizing the gap between the lumbar spine and the spine coil. Furthermore, a strap fastened around a small rectangular pad was used across the abdomen to facilitate even closer contact with the spine coil, yet without causing any discomfort.

### Image Analysis Protocol

**Fig 2:**
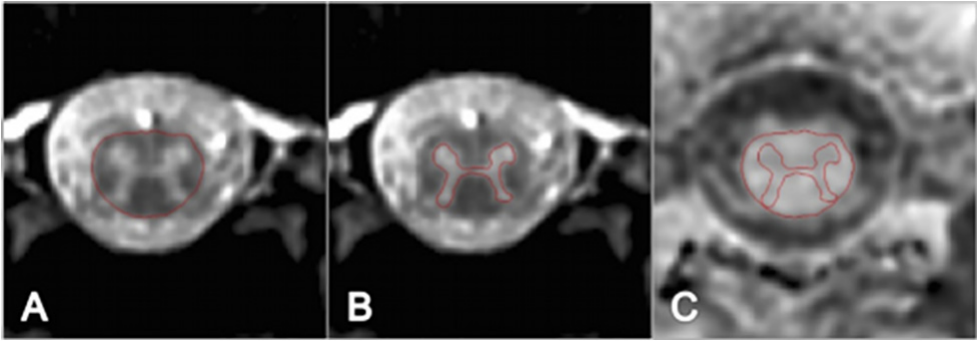
A) Cord cross-sectional area (CSA) contour identified using the active surface model tool (ASM) B) GM area contour identified using manual segmentation. C) CSA and GM area contours are shown overlaid onto the MTR-map following linear co-registration.

Using the linear registration toolkit in FSL (http://www.fmrib.ox.ac.uk/fsl/) with schedule file ‘sch2D_6dof’ and all other options set to default, the MT-off and MT-on volumes were co-registered independently to the 3D-FFE high-resolution structural scan, prior to calculation of the MTR-map; this was necessary since the MT-on and MT-off images were not acquired interleaved therefore motion could have occurred between the two scans, and also to account for possible changes in the cord position between the acquisition of the 3D-FFE sequence and the MT-on and MT-off data. Mean CSA and MTR measurements were obtained from a 15 mm section (i.e. 3 slices) through the widest segment of the LSE as follows [24]: using the active surface model (ASM) segmentation method in JIM 6.0 (Xinapse systems, www.xinapse.com) [27], seed points were manually positioned in the centre of the cord on each slice of the 3D-FFE structural scan and the cord area was identified and recorded. The slice with the largest CSA was subsequently selected along with the two adjacent slices in order to calculate the mean LSE-CSA for that section (Fig 2A). From the same section, GM was segmented using JIM 6.0 and the mean area (LSE-GM-CSA) was calculated as previously described [24] (Fig 2B).

Binary masks of GM and WM were obtained (the WM mask was generated by subtracting the GM mask from the total cord mask) and applied to the MTR map to calculate mean MTR values within GM (GM-MTR) and white matter (WM-MTR) (Fig 2C). In order to ensure that GM- and WM-MTR were not significantly affected by partial volume—i.e. cerebrospinal fluid (CSF) contamination, inaccuracies at the WM and GM boundaries and possible subtle registration errors—the WM and GM masks were first eroded using FSL (S1 Fig).

### Reproducibility Assessment

The reproducibility of the MTR parameters was assessed by performing a ‘scan-rescan test’ on five out of ten healthy volunteers with a minimum of seven days (and a maximum of 14 days) in between the first and the second visits. One experienced rater analysed all the data. In order to demonstrate the intra-rater reproducibility of the GM and WM segmentation method, the same rater re-analysed all the data from the 5 volunteers’ first visit twice; the analysis was performed on separate occasions with a minimum of 1 week between each analysis. Inter-rater reproducibility was assessed by employing a second rater to analyse the data from the 5 volunteers’ first visit. The two raters were unaware of the results of each other.

### Statistical Analysis

For the scan-rescan assessment of the MTR acquisition and the intra- and inter-rater reproducibility of the segmentation method, the coefficient of variation (COV) was calculated using the mean and standard deviation from the repeated measures and the equation COV = [SD/mean] × 100%. The intra-rater segmentation results were further evaluated using the Dice similarity coefficient (DSC) [24, 28].

Possible differences in MTR values between GM and WM were assessed using a paired t-test, after checking the normality of the data. Statistical significance was accepted at *p*<0.05.

## Results

Mean (± SD) LSE-CSA of the 15 mm section studied (i.e. 3 slices) across 10 healthy subjects was 59.3 (± 8.4) mm^2^ and mean (± SD) LSE-GM-CSA was 17.0 (± 3.1) mm^2^. The mean intra- and inter-rater % COV for measuring the LSE-CSA were 0.8% and 2.3%, respectively and for measuring the LSE-GM-CSA were 3.8% and 5.4%, respectively. Mean (± SD) DSC for a single rater (intra-rater) of the LSE-CSA was 0.98 (± 0.01) and of the LSE-GM-CSA was 0.92 (± 0.01).

Tissue-specific MTR values within these volumes were determined from the calculated MTR maps. Mean (± SD) WM-MTR was 43.2 (± 4.4) and the mean (± SD) GM-MTR was 40.9 (± 4.3). The mean scan-rescan % COV for the WM-MTR was 4.6% and for the GM-MTR was 3.8%. A paired t-test showed a statistically significant difference between the WM-MTR and GM-MTR (*p*<0.0001).

**Fig 3:**
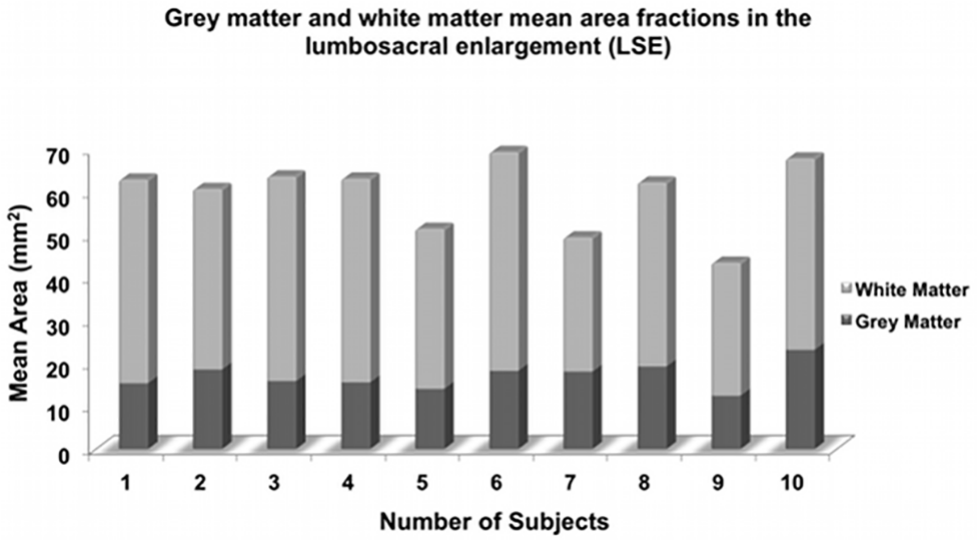
Stacked plot diagram showing grey matter (GM) and white matter (WM) mean area fractions measured in a 15 mm section through the lumbosacral enlargement (LSE) in 10 healthy subjects.

Table 1 summarizes the main results of the study. Fig 3 shows a stacked plot diagram of tissue-specific CSA measurements in all 10 healthy subjects. S1 Dataset contains all the raw data for the study.

**Table 1:** Cross-sectional area (CSA) and magnetisation transfer ratio (MTR) measurements in the lumbosacral enlargement (LSE) of 10 healthy subjects.

Measurement	Mean	SD	Scan-rescan (%COV)	Intra-rater (%COV)	Inter-rater (%COV)	DSC
LSE-CSA (mm^2^)	59.3	8.4	-	0.8	2.3	0.98
LSE-GM-CSA (mm^2^)	17.0	3.1	-	3.8	5.4	0.92
WM-MTR	43.2	4.4	4.6	0.8	0.9	-
GM-MTR	40.9	4.3	3.8	0.4	0.8	-

Abbreviations:- LSE-CSA: Lumbosacral enlargement cross-sectional area; LSE-GM-CSA: Lumbosacral enlargement grey matter cross-sectional area; WM-MTR: White matter magnetisation transfer ratio, GM-MTR: Grey matter magnetisation transfer ratio; DSC: Dice similarity coefficient.

## Discussion

In this study, the feasibility of obtaining tissue-specific (i.e. GM and WM) MTR measurements within the LSE was investigated for the first time using a commercially available 3T MR system, software and hardware and by addressing a number of technical considerations. To obtain tissue-specific quantitative measurements within the lower SC, first a reliable way to depict these tissue types was essential. Based on a recently published report, high-resolution images of the LSE were acquired within the MTR imaging protocol and thereafter used for image segmentation [24]. The effectiveness of the segmentation method was evaluated and the results were found to be in agreement with the previous report, with favorable intra-rater COV values of 3.8% for measuring the LSE-GM-CSA and 0.8% for the LSE-CSA, as compared to 8% and 2%, respectively. Inter-rater COV values were also in agreement with 5.4% for measuring the LSE-GM-CSA and 2.3% for the LSE-CSA, as compared to 8.6% and 2.5%, respectively. In addition, the DSC of the LSE-CSA and LSE-GM-CSA in this study were 0.98 and 0.92, respectively as compared to 0.97 and 0.89, respectively in the previous report. These results confirmed the reliability of the segmentation method, which was subsequently used to facilitate the tissue-specific MTR measurements in this study.

The MTR acquisition was set up with imaging parameters to match the structural acquisition as closely as possible, albeit with some necessary modifications. Due to SNR restrictions, it was not possible to achieve identical in-plane resolution to the structural scan (i.e. 0.5 x 0.5 mm^2^), requiring post-acquisition interpolation (i.e. in k-space) of the data acquired at a 0.75 x 0.75 mm^2^ resolution. SNR restrictions may be addressed either by using a larger voxel size, longer acquisition times or with the use of dedicated RF coil designs, but such options were either unsuitable or unavailable in this particular case. Furthermore, the physiological motion arising from major blood vessels, the kidney and the bowel function, often requires the use of regional saturation (REST) slabs to compensate. However, such measures of physiological motion-related image artifact reduction involve the use of additional RF pulses within the pulse sequence, with concomitant negative effects on the measurable MTR. To partly account for this, the MTR sequence was used with a radial acquisition profile and the fold-over direction set in the foot/head (FH), therefore alleviating the need for REST slabs. However, using radial acquisitions, aliasing artifacts are often encountered and must be accounted for whenever possible. For this reason, a much larger imaging volume was used than the anatomical region of interest, and the edge slices that were affected the most were subsequently discarded. Of course, this also meant that the scanning time was increased and future pulse sequence developments should try to address such limitation. Additional sources of physiological motion-related artifacts in spinal cord imaging may include CSF pulsation, though less concerning in this case considering the amplitude of pulsation is considerably lower in the lumbar spine than in the cervical and thoracic spine [29, 30].

In this study, the high resolution structural and MTR acquisition protocol for studying the LSE was optimized using a clinical 3T MR system. Higher magnetic field offers better SNR and enables more efficient acquisition with improved conspicuity of structures than lower magnetic field MR systems [31, 32]. In the spinal cord, the potential for higher resolution imaging is particularly important considering the small size of the structure and the poorly defined boundary between GM and WM. The use of lower magnetic field systems to obtain similar measurements as in this study would require considerably longer scan time and possibly extending beyond clinically acceptable times. However, in applications where such high resolution is not required, excellent image contrast and reliable MTR measurements may be obtained by using both high and low magnetic field MR systems [31].

The structural images were co-registered with the MTR maps using linear registration, first by registering the MT-off and MT-on volumes independently to the structural scan, prior to calculation of the MTR-map. Despite matching the imaging parameters of the acquisitions as closely as possible, it is likely that the use of a radial acquisition profile for MTR imaging may have hampered the registration process due to the differences in data reconstruction between Cartesian and radial profiles [26]. Nevertheless, to partly account for such potential inaccuracies, the GM and WM masks were eroded prior to the calculation of MTR values and therefore such effects, as well as CSF contamination and inaccuracies at the WM and GM boundaries, are unlikely to have confounded the measurements considerably.

In this study, *in vivo* WM-MTR and GM-MTR normative values in 10 volunteers measured from the total volumes extracted from a 15 mm section of the LSE are reported for the first time. The significant difference in MTR values observed between GM and WM (p<0.0001), is likely to reflect a difference in myelin content between these tissue types. However, the absolute MTR measure reflects a complex combination of various biological factors and is also highly sequence- and MR system-dependent, therefore the MTR values obtained in this study cannot be directly compared with those obtained using a different sequence or at another centre. Quantitative MT (qMT) imaging provides more fundamental parameters relating to tissue structure and future investigations using this technique may allow further insight into differences between GM and WM in the lower SC.

Obtaining tissue-specific MTR measurements within the LSE may have potential clinical applications in the evaluation of patients where a lower SC lesion is suspected. Spinal cord injury [33–38], and MS [39, 40] may affect the lower SC, resulting in lower limb weakness and LUT, sexual and bowel dysfunction. In neurodegenerative conditions such as multiple system atrophy (MSA) selective degeneration occurs in the lower SC, specifically the Onuf’s and parasympathetic nuclei of the sacral cord resulting in urinary retention and changes of reinnervation in the anal sphincter muscle as detected by concentric needle EMG [41, 42]. *Ex vivo* investigations have provided evidence of somatic and motor neurons loss in the lower SC in MSA [43], and pathology in the LSE is probably the main cause of LUT dysfunction. However, it is not possible using the current protocol to identify with certainty the regions of the LSE from which the somatic, parasympathetic and sympathetic innervation for the LUT arise [23]. Routine clinical MRI sequences are often insensitive to detect such changes however, and the use of quantitative imaging measures in the lower SC (LSE) would help to evaluate intrinsic pathological abnormalities *in vivo* with the potential for clinical applications.

Some of the limitations of the present study, both in terms of image acquisition and analysis, have already been mentioned. However, when evaluating these methods in the context of clinical utility, additional obstacles may be considered that have not been encountered in this pilot study of healthy volunteers. In terms of the acquisition, the long scans required to depict GM/WM and obtain reliable MTR measurements within the LSE may result in image artifacts due to involuntary patient motion; for this reason, particular attention to the immobilization technique is required, especially in neurological conditions which are associated with involuntary lower limb movement. The MTR acquisition presented in this study may benefit from further refinement in the future in order to reduce the acquisition time by employing more efficient coverage of smaller anatomical regions such as the LSE. In terms of image analysis, the presence of lesions or atrophy in the spinal cord may impose further challenges, such as difficulties in GM/WM segmentation and subsequent MTR measurements; in addition, the COV values observed in healthy volunteers for segmenting GM in the LSE will certainly affect the ability to detect subtle changes in disease state therefore this method may require a large sample size or to asses damage that has progressed considerably. Future work will therefore be directed at reducing (or removing altogether) operator input, for example through the use of lumbar spinal cord templates, or other fully-automated image analysis methods depending on the specific application.

In summary, the results of this study show that it is possible to obtain *in vivo* tissue-specific MTR measurements within the LSE using a clinical MR system at 3T. This pilot study provides a solid foundation for future investigative studies involving patients with neurological disorders affecting the LSE region of the SC.

## Supporting Information

S1 Dataset Raw data. (XLSX)

S1 Fig A) Example of grey matter segmentation (contour shown in red) B) pixels within grey matter with minimal or no partial volume (green points) maybe used to calculate MTR. (TIFF)
