# Supplementary figures and images for: Grey and White Matter Magnetisation Transfer Ratio Measurements in the Lumbosacral Enlargement: A Pilot In Vivo Study at 3T
Source: PLoS One. 2015 Jul 31;10(7):e0134495. doi: 10.1371/journal.pone.0134495 (PMC4521783; doi:10.1371/journal.pone.0134495)

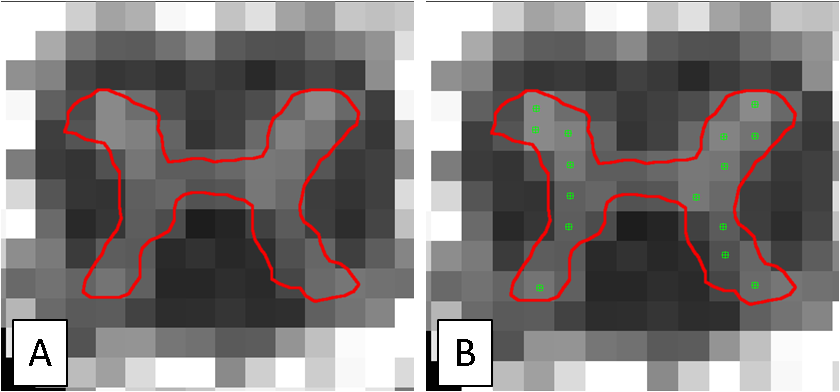

Supplement: S1 Fig — (TIFF) [file pone.0134495.s002.tiff]
